# Early Selection for Smut Resistance in Sugarcane Using Pathogen Proliferation and Changes in Physiological and Biochemical Indices

**DOI:** 10.3389/fpls.2016.01133

**Published:** 2016-07-28

**Authors:** Yachun Su, Zhuqing Wang, Liping Xu, Qiong Peng, Feng Liu, Zhu Li, Youxiong Que

**Affiliations:** Key Laboratory of Sugarcane Biology and Genetic Breeding, Ministry of Agriculture, Fujian Agriculture and Forestry UniversityFuzhou, China

**Keywords:** sugarcane, smut, disease resistance, copy number, physiological and biochemical indices, principal component analysis, subordinate function analysis

## Abstract

Sugarcane smut disease, caused by *Sporisorium scitamineum*, significantly decreases yield and use of resistant cultivars is the most cost-effective measure for disease control. Current field testing methods for identification of smut resistance are time-consuming and hindered by environmental variability. Our goal was to develop an efficient and reliable resistance identification technique that is rapid, performed in a controlled environment, and stable. Nine sugarcane cultivars with different phenotypic resistance levels were selected. TaqMan quantitative real-time polymerase chain reaction analysis was performed to measure copy number changes of smut pathogen in sugarcane buds at 0–7 days after needle puncture inoculation. There was a positive correlation between time after inoculation and the amount of smut pathogen in the sugarcane bud. This reached a peak value on 7 days, and the copy number of *S. scitamineum* increased in the following order: YZ03-258, FN40, YZ01-1413, GT02-467, ROC22, YT96-86, YZ03-103, FN39, LC05-136. After smut pathogen inoculation, differences in the physiological and biochemical indices of the nine cultivars were observed. Peroxidase, ascorbate peroxidase, catalase, superoxide dismutase, β-1,3-glucanase, and malondialdehyde were grouped into three main components, and the cumulative contribution rate was 80.177%, revealing that these are useful physiological and biochemical indicators of smut resistance. Subordinate function analysis indicated that the levels of smut resistance of the nine genotypes were (high to low): YZ03-258, FN40, YZ01-1413, GT02-467, ROC22, YZ03-103, YT96-86, FN39, LC05-136, which is similar to the results from copy number determination of smut pathogens. The results suggest that after artificial needle inoculation, rapid identification of physiological resistance to sugarcane smut was achieved based on copy number increases in the sugarcane smut pathogen and the physiological and biochemical changes in sugarcane bud during the early phase of infection.

## Introduction

Sugarcane (*Saccharum* spp.) is the most important sugar crop. Its sucrose accounts for 80% of the total sugar production worldwide, and 92% of the total sugar production in China. Sugarcane smut, caused by *Sporisorium scitamineum*, is one of the most severe fungal diseases affecting sugarcane production, and all producing countries have developed protocols for the prevention and control of this disease (Sundar et al., 2012). Cultivation of smut-resistant sugarcane cultivars is the most economical and effective measure for disease prevention and control (Scortecci et al., 2012; Sundar et al., 2012).

Sugarcane smut resistance is influenced by three major factors: sugarcane genotype, the pathogen, and the environment. Although difficult, accurate identification of smut resistant sugarcane genotypes is essential to breeding disease-resistant cultivars. Disease resistance is typically evaluated by dipping a sugarcane stalk into a solution of teliospores and planting the inoculated stalk in the field, followed by observation of disease incidence. Resistance is also studied using multiple epidemiological parameters (Xu and Chen, 2001; Singh et al., 2005; Que et al., 2006; Olweny et al., 2008). The field resistance identification technique generally requires at least 6–18 months, i.e., one plant-crop season and the following ratoon, to accurately determine resistance. Comprehensive analysis of multiple epidemiological parameters in the crop-growing season can help overcome the incompleteness of the evaluation results derived from a single parameter (Lloyd and Pillay, 1980; Xu and Chen, 2000), but an objective evaluation is still difficult due to interfield variation and interaction effects. As Burner et al. (1993) suggested that the geographical environment has a significant impact on sugarcane resistance phenotypes. Because cross-breeding of sugarcane occurs over large-scale areas and involves long time periods, it is a tedious process. The establishment of a smut resistance identification system with controllable conditions, a short cycle, and high efficiency is needed to expand the screening of candidate breeding materials. It is particularly important to establish a rapid and stable evaluation method to evaluate sugarcane smut disease (Churchill et al., 2006; Singh et al., 2014). Su et al. (2013a) described a TaqMan quantitative real-time polymerase chain reaction (qRT-PCR) detection system for sugarcane smut pathogens. Differences in the rates of pathogen proliferation after artificial inoculation of one smut-resistant and one susceptible sugarcane genotypes were observed, and the copy number of pathogens in the susceptible cultivar was significantly higher than that of the resistant cultivar (Su et al., 2013a). This suggested that the TaqMan qRT-PCR system could be useful in early stage identification of smut-resistance in sugarcane cultivars. However, this finding requires further verification related to the interaction between the pathogen and sugarcane genotypes.

Once plants are infected by pathogens, the cell structure of plants is destroyed, and the level of intracellular reactive oxygen species (ROS) increases. This induces a series of physiological and biochemical metabolic reactions in the host plant. Among these reactions, the activity of defense enzymes plays an important role in plant physiological defense during early stages of pathogen invasion (Blokhina et al., 2003). Sugarcane buds serve as the invasion route of smut pathogens. Sugarcane resists smut invasion in two ways: morphological resistance (i.e., defense is conducted via bud-scale barrier or bud exudates) and physiological and biochemical resistance (i.e., across bud-scale barrier, plant damage due to pathogen stress is regulated by the interaction between sugarcane tissues and smut pathogens; Waller, 1970; Dean, 1982; Whittle and Walker, 1982; Chao et al., 1989; Xu and Chen, 2000). Physiological and biochemical resistance in sugarcane involves various internal defense responses which are triggered after the pathogen penetrates the bud-scale barrier. These include the synthesis of flavonoids and altered concentrations of phenolic compounds, physiological enzymes, and glycoside substances. Lignin concentration may increase as well as increased production of glycoproteins, salicylic acid, and polyamines (Lioyd and Naidoc, 1983; Gong et al., 1996; Shrivastava et al., 2003; Mo, 2012; Sundar et al., 2012; Karen et al., 2013). Peroxidases, a large class of plant enzymes, also play an important role in plant disease resistance in numerous species (Chassot et al., 2007; Ebrahim et al., 2011).

Peroxidase (POD) belongs to the pathogen-associated protein 9 subfamily, and the excessive accumulation of ROS causes changes in plant peroxidase activity and gene expression levels. POD expression is closely related to plant disease resistance (Hiraga et al., 2001). Wen et al. (2006) measured the POD activities of four resistant and three susceptible *Setaria italica* cultivars at different growth stages and found that the POD activity of resistant cultivars was significantly higher than in susceptible ones, indicating that POD levels could potentially be used as a genetic marker for resistance evaluation. These results are similar to findings of Xu et al. (1994) and Xing (2013). Superoxide dismutase (SOD) is an enzyme containing metal cofactors that are specific for scavenging superoxide anions in plants. SOD uses free radicals as substrates, and this is the first-line of defense against various biotic and abiotic stresses in plants (Dat et al., 2000). Ascorbate peroxidase (APX) is a key enzyme in plants that scavenges H_2_O_2_. APX has a high affinity with ascorbic acid (AsA), using a small number of its electrons to reduce H_2_O_2_ to H_2_O, which reduces intracellular H_2_O_2_ and prevents the damage from ROS (Sato et al., 2001). Catalase (CAT) is a key enzyme that maintains intracellular ROS balance and plays an important role in scavenging the H_2_O_2_ that is produced during mitochondrial electron transport and the oxidation of fatty acids (Su et al., 2014). β-1,3-glucanase is active against fungal diseases, and plays a direct role in biological and chemical defenses in plants (Su et al., 2013b). Malondialdehyde (MDA) is a non-enzymatic and physiologically active substance in plants, and derives from lipid peroxidation in cell membranes. Its mass production enhances biofilm damage and inhibits the activities of cell protective enzymes. The content of MDA in plant tissues reflects the degree of incurred damage (Zhang B.Q. et al., 2015). Zhang J.R.F. et al. (2015) studied the relationship between the physiological and biochemical changes and disease resistance in *Panicum miliaceum* after smut infection, and found that with artificial inoculation, the increase in the MDA content is relatively small. However, the protective enzyme system and the pathogenesis-related proteins (e.g., POD, SOD, β-1,3-glucanase, and chitinase) of cultivars with high levels of resistance are resistant to the damages inflicted by the pathogen. The above studies indicate that the activities of these physiological enzymes and the contents of biochemical substances can provide a better understanding of plant resistance mechanisms and the extent of damage to the membrane systems. They can also help identify genotypic resistance. The application of these enzymes during the early selection stage of smut disease-resistant sugarcane clones has not been studied.

The combination of principal component analysis (PCA) and subordinate function can convert each index to independent factors that can be compared with each other while maintaining the original information. A comprehensive evaluation value for the resistance of each cultivar is obtained, providing a more thorough evaluation of plant resistance (Churchill et al., 2006; Han et al., 2006; Zhang B.Q. et al., 2015). PCA has been widely used to study sugarcane cold resistance (Zhang B.Q. et al., 2015), *Medicago sativa* drought resistance (Han et al., 2006), and the antioxidant activity of chewing cane (Lin et al., 2007). However, its application to studies on sugarcane resistance to smut disease is unexplored. Therefore, the present study used nine sugarcane cultivars, with various levels of smut resistance, field planted after artificial inoculation with smut pathogen. The copy number of pathogens in the sugarcane buds after inoculation was measured by TaqMan qRT-PCR, and the correlation between phenotype resistance and proliferation of smut pathogens was analyzed. Based upon the changes in the activities of POD, SOD, APX, CAT, MDA, and β-1,3-glucanase in sugarcane buds, PCA was used to screen the physiological and biochemical indicators that are closely related to smut resistance and the resistance was comprehensively evaluated using subordinate function. The study goal was to establish a rapid, accurate, and reliable technique for evaluating sugarcane smut resistance during early breeding stages.

## Materials and Methods

### Plant Materials and Treatments

A total of nine sugarcane (*Saccharum* spp. hybrid) cultivars (**Table 1**; Supplementary Table S1) were provided by the Key Laboratory of Sugarcane Biology and Genetic Breeding, Ministry of Agriculture (Fuzhou, China). The scientist at the China Agricultural Research System used a teliospore suspension of *S. scitamineum* (5 × 10^6^ spores/mL) to dip inoculate the sugarcane stalks for 10 min and incubate at 25–28°C in 100% humidity for 1 day. This was followed by field planting to determine smut resistant phenotypes (Chao et al., 1990; Xu and Chen, 2001). The nine sugarcane cultivars included four resistant (YZ03-258, YZ01-1413, YT96-86, and LC05-136), three medium susceptible (GT02-467, ROC22, and FN39), and two susceptible (YZ03-103 and FN40) cultivars. The incidence of sugarcane smut in the nine genotypes was in the following order: YZ03-258 < YZ01-1413/LC05-136 < YT96-86 < GT02-467 < ROC22 < FN39 < YZ03-103 < FN40 (personal communication with Yingkun Huang).

**Table 1:** Nine sugarcane varieties with different resistance to smut after identification in the field.

No.	Variety	Sugarcane smut incidence (%)	Resistance classification	Resistance rating
1	YZ03-258	0.0	1	HR
2	YZ01-1413	4.5	2	R
3	LC05-136	4.5	2	R
4	YT96-86	5.0	2	R
5	GT02-467	14.1	5	MS
6	ROC22	14.5	5	MS
7	FN39	17.0	5	MS
8	YZ03-103	30.6	6	S
9	FN40	33.1	6	S

*The smut resistance ratings in sugarcane according to Chao et al. (1990) and Xu and Chen (2001) are as follows: 0–3%, HR; 4–6%, R; 7–9%, R; 10–12%, MR; 13–25%, MS; 26–35%, S; 36–50%, S; 51–75%, HS; 76–100%, HS. HR, highly resistance; R, resistance; MR, moderate resistance; MS, moderate susceptibility; S, susceptibility; HS, highly susceptibility.*

*The data of sugarcane smut incidence come from personal communication with Yingkun Huang.*

Teliospores of sugarcane smut, provided by the Key Laboratory of Sugarcane Biology and Genetic Breeding, used as the inoculum source were dried and stored at 4°C until use. Sugarcane stalks from all nine cultivars with relatively consistent phenotypes were selected, and inoculation was performed according to Su et al. (2014). The sugarcane stalk was cut into two-bud setts, soaked in running-water for 1 day, and incubated at 32°C to allow the buds to grow to 2 cm, under a 16:8-h light-dark photoperiod. A smut spore suspension of 5 × 10^6^ spores/mL (with 0.01% Tween-20, v/v) was inoculated by puncturing the sugarcane bud with a needle. The bud inoculated with sterile distilled water (with 0.01% Tween-20, v/v) was the control. The punctured material was cultured at 28°C in a 16:8-h light-dark photoperiod. Sugarcane buds were collected on 0, 1, 3, and 7 days after inoculation, and fixed in liquid nitrogen in a freezer at -80°C until use. Among these, five mixed sugarcane bud samples were used to determine the copy number of smut pathogens, and five mixed sugarcane bud samples were used to measure physiological and biochemical parameters. Two biological replicates were prepared for the experiment, and three technical replicates were used for each biological replicate.

### DNA Extraction

Sugarcane genomic DNA was extracted using the hexadecyl trimethyl ammonium bromide (CTAB) method of Yao et al. (2005). The spores of sugarcane smut pathogens were cultivated using the method of Que et al. (2004), and the sodium dodecyl sulfonate (SDS) method (Que et al., 2004) was used to extract genomic DNA from the mycelia. Genomic DNA was then treated with 100 μg/mL RNase A in a water bath at 37°C for 0.5 h to remove RNA. After the DNA size was determined using 1% agarose gel electrophoresis, sample concentration and purity were measured by NanoVue plus (GE Healthcare, Piscataway, NJ, USA). The DNA samples were stored at -20°C until analysis.

### Measurement of the Copy Number of Sugarcane Smut Pathogens

The TaqMan qRT-PCR system was used for the detection of sugarcane smut pathogens. It was completed according to Su et al. (2013a) using the sugarcane smut *bE* gene which was the target gene used to measure pathogen copy number in all sugarcane samples. The primers used in the analysis were bEQ-F: 5′-TGAAAGTTCTCATGCAAGCC-3′ and bEQ-R: 5′-TGAG AGGTCGATTGAGGTTG-3′, and the sequence of the TaqMan probe was 5′-FAM-TGCTCGACGCCAATTCGGAG-TAMRA-3′. The *bE* recombinant plasmid pMD18-T-bE, constructed using the method described by Su et al. (2013a), was the standard positive control, genomic DNA of healthy sugarcane tissue culture plantlets was used as the negative control, and sterile water was used as the blank. The components of quantitative PCR reaction system were as follows: 12.5 μL of 2×*Taq*Man Universal Master Mix, 1.0 μL each of upstream (10 μmol/L bEQ-F) and downstream (10 μmol/L bEQ-R) primer, 0.2 μL of TaqMan probe (10 μmol/L), and 1.0 μL of DNA template (500 ng/μL); ddH_2_O was added up to 25 μL. PCR reaction conditions were as follows: 50°C for 2 min; followed by 40 cycles of 95°C for 10 min, 95°C for 15 s, and 60°C for 1 min.

We used the formula from Jamnikar and Toplak (2012) in which MW = base number × 660 dalton/bp and copies/mL = 6.02 × 10^23^ × (concentration g/mL)/(MW g/mol), to calculate the DNA copy number of sugarcane smut pathogens. Because the length of the *bE* gene fragment was 459 bp, the DNA copy number of the recombinant plasmid pMD18-T-bE at a concentration of 100 ng/μL was 1.987 × 10^11^ copies/μL. A 10-fold serial dilution was performed on this plasmid DNA, and five plasmid samples with final concentrations ranging from 10^-4^ to 10^-8^ copies/μL were used as templates for TaqMan qRT-PCR analysis, followed by plotting the standard curve.

### Determination of the Physiological and Biochemical Indices of Sugarcane

To extract the crude enzyme, approximately 1 g of sugarcane buds was placed in a pre-chilled mortar. After adding a small amount of quartz sand, an appropriate amount of polyvinyl polypyrrolidone (PVP), and 10 mL of pre-cooled 0.05 mol/L phosphate buffer (pH 7.8), the sample was homogenized by rapid grinding, and immediately transferred into 15 mL centrifuge tubes. Centrifugation was 5,000 *g* for 15 min at 4°C. The supernatant was transferred into a new centrifuge tube for a second centrifugation under the same conditions. The resulting supernatant was collected as a crude enzyme solution for the test, and stored at 4°C until use. Analysis of POD and SOD enzyme activity was conducted using the methods of Mo (2012) and Xing (2013) with minor modifications (Supplementary Data Sheet S1), respectively. Analysis of APX activity was based on the method of Chen (2012), wherein the concentration of H_2_O_2_ was 0.1 mol/L. Measurements of β-1,3-glucanase, MDA, and CAT activities were conducted according to methods of Su et al. (2013), Xing (2013), and Su et al. (2014) respectively.

### Data Processing and Statistical Analysis

Data processing, PCA, and subordinate function calculation were conducted using Excel (Office 2013) and SPSS (version 17.0). The physiological and biochemical indices measurements were used to calculate the mean values of the data for the control and treatment groups. The formula-coefficient of resistance (%) was calculated using the following equation: coefficient of resistance (%) = (measured value for treated sample/measured value for control) × 100%, was used to convert the index data to calculate the resistance coefficients. Correlation analysis provided the correlation coefficient matrix for each physiological and biochemical index. The characteristic root and eigenvector of the index-related matrix, as well as the contribution rate and cumulative contribution rate of each principal component were calculated, a principal component equation was established, and the common factor analysis was performed (Churchill et al., 2006; Han et al., 2006). The subordinate function value for each index was calculated using the following equation: U(X_i_) = (X_i_ - X_min_)/(X_max_ - X_min_) (*i* = 1, 2, 3,…n), where *X*_i_ is the measured index value, and *X*_min_ and *X*_max_ are the minimum and maximum values of one given index for all tested materials. Weights were calculated using the following equation: W_j_ = P_j_/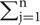 P_j_ (*j* = 1, 2, …, n), where W*_j_* is the importance of *j*th common factor in all common factors, and *P_j_* is the contribution rate of the *j*th common factor for each plant. The subordinate function value and the weight value were used to calculate the comprehensive evaluation value of sugarcane smut resistance using the following equation: D(X) = ∑ _j=1_[U(X_j_) × W_j_] (*j* = 1, 2, …, n) (Zhang B.Q. et al., 2015).

## Results

### Proliferation Rate of Pathogens in Sugarcane Buds after Inoculation

**FIGURE 1:**
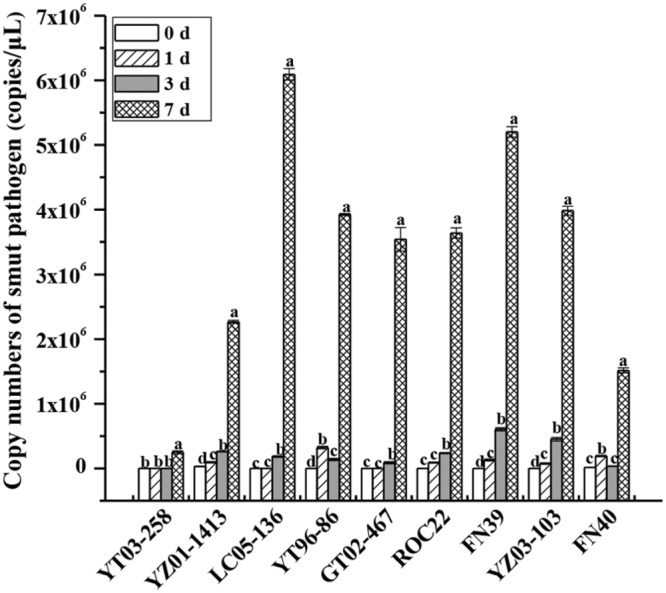
**Copy number variations in the smut pathogen with different sugarcane genotypes as indicated by TaqMan qRT-PCR analysis.** A smut spore suspension of 5 × 10^6^ spores/mL was inoculated by puncturing the sugarcane bud with a needle. The bud samples were collected at the early stages of 0, 1, 3, and 7 days and analyzed by TaqMan qRT-PCR technique. Copy numbers of smut pathogen were calculated with the equation of the linear regression line. Error bars were represented as standard error. YZ03-258, YZ01-1413, YT96-86, and LC05-136 were smut resistant cultivars. GT02-467, ROC22, and FN39 were medium susceptible cultivars. YZ03-103 and FN40 were susceptible cultivars.

Based on the identification of field smut resistance, nine different sugarcane genotypes were used to analyze the proliferation rate of smut pathogens in sugarcane buds after inoculation. TaqMan qRT-PCR analysis indicated that the proliferation rate of *S. scitamineum* gradually increased with increasing inoculation time (0–7 days) although there were differences in the proliferation rate among the genotypes (**Figure 1**). At the 0 and 1 day time points, no smut pathogens were detected in YZ03-258, LC05-136, and GT02-467, and the copy number of the other sugarcane genotypes slowly increased. At 3 days, the copy number of pathogens in YZ03-258 remained at the control level, while copy numbers in YT96-86 and FN40 decreased relative to 1 day. At 3 days, copy numbers in the medium susceptible genotypes ROC22 and FN39 and susceptible genotype YZ03-103 were higher than those of the other sugarcane genotypes. At 7 days, the number of smut pathogens in the nine genotypes had significantly increased and reached a peak, but the proliferation rate of resistant genotypes YZ03-258 and YZ01-1413 were lower than the other sugarcane genotypes. Copy numbers of smut pathogens, in an ascending order, were YZ03-258 < FN40 < YZ01-1413 < GT02-467 < ROC22 < YT96-86 < YZ03-103 < FN39 < LC05-136. Comparison of earlier findings on the incidence of field smut disease (in ascending order: YZ03-258 < YZ01-1413/LC05-136 < YT96-86 < GT02-467 < ROC22 < FN39 < YZ03-103 < FN40) showed that, except for LC05-136, YT96-86, and FN40, the copy numbers of smut pathogens were generally in the same order as the incidences of field smut disease among the six sugarcane genotypes. Cultivars with a high level of field phenotypic resistance to smut disease had a relatively low pathogen proliferation rate after smut infection. This result suggests that quantification of spore copy number can be used to select smut resistant sugarcane clones as early as 7 days after infection.

### Physiological and Biochemical Responses of Sugarcane after Smut Pathogen Stress

Changes in six physiological and biochemical indices, defense enzymes (POD, SOD, APX, and CAT), pathogenesis-related protein (β-1,3-glucanase) as well as MDA (directly related to cell peroxidation) were measured (Supplementary Table S2). A series of changes occurred in all the physiological and biochemical indicators of different sugarcane genotypes in the presence of smut pathogen stress. Significant differences in the magnitude of the changes in the physiological and biochemical indices among different sugarcane genotypes and the same sugarcane genotype at different time points of smut pathogen stress were seen. Therefore, the use of a single index to evaluate the level of sugarcane smut resistance can lead to different conclusions regarding resistance.

### Correlation Analysis and PCA of All Physiological and Biochemical Indices of the Sugarcane Genotypes

The measured physiological and biochemical indices for the control and treatment groups were calculated to obtain a mean value of each index, and then the resistance coefficients of each indicator were calculated (**Table 2**). Correlation analysis produced a correlation coefficient matrix for each indicator (**Table 3**). Different degrees of correlation among the six physiological and biochemical indices the nine genotypes were observed, and some physiological and biochemical indices were significantly correlated. For example, a significant positive correlation between POD and β-1,3-glucanase as well as between APX and β-1,3-glucanase was found (*P* ≤ 0.05), indicating that a higher POD or APX activity represents an increase in β-1,3-glucanase activity, however, the correlations among other indicators were not significant. Thus, the use of a single indicator to evaluate sugarcane smut resistance appears to be inadequate.

**Table 2:** Disease-resistance coefficients of physiological and biochemical indices in different sugarcane varieties.

Variety	Disease-resistance coefficients (%)					
	POD	APX	CAT	SOD	β-1,3-glucanase	MDA
YZ03-258	155 b	169 a	205 b	280 c	150 a	56 g
YZ01-1413	133 c	132 d	148 d	182 d	116 c	446 a
YT96-86	119 e	48 i	70 g	312 a	81 d	62 f
LC05-136	77 g	64 h	112 e	132 g	85 d	85 d
GT02-467	192 a	88 e	109 e	138 f	150 a	-37 h
ROC22	126 d	136 c	88 f	166 e	109 c	-135 c
FN39	66 h	71 g	238 a	131 g	71 e	-71 e
YZ03-103	113 f	84 f	95 f	116 h	87 d	147 b
FN40	127 cd	148 b	160 c	296 b	131 b	59 fg

*Values in the same column with the different letter were significantly different (*P-*value < 0.05) calculated using one-way analysis of variance (ANOVA) followed by multiple Duncan tests. POD, peroxidase; APX, ascorbate peroxidase; CAT, catalase; SOD, superoxide dismutase; MDA, malonaldehyde.*

**Table 3:** Correlation matrix of test indices.

Indexes	POD	APX	CAT	SOD	β-1,3-glucanase	MDA
POD	1.000					
APX	0.442	1.000				
CAT	-0.212	0.358	1.000			
SOD	0.246	0.345	0.044	1.000		
β-1,3-glucanase	0.873^∗^	0.726^∗^	0.132	0.301	1.000	
MDA	0.037	0.128	-0.018	0.056	0.024	1.000

*^∗^Denotes significant differences at 0.05 level. POD, peroxidase; APX, ascorbate peroxidase; CAT, catalase; SOD, superoxide dismutase; MDA, malonaldehyde.*

The PCA results showed that after filtering the correlation coefficients by phase rotation (**Table 4**), the cumulative contribution rate of three principal factors (represented by 1, 2, and 3, respectively) was >80%. This indicated that these three components reflect 80.177% of the information on the smut resistance of nine genotypes (Zhang B.Q. et al., 2015). According to the eigenvalue of each factor, the first principal component included POD, APX, CAT, and SOD, the second principal component was β-1,3-glucanase, and the third principal component was MDA. The value of the contribution rate reveals the relative importance of various physiological and biochemical indices of sugarcane. The three above-mentioned independent comprehensive indicators could be used to evaluate smut resistance in sugarcane.

**Table 4:** Results of principal component analysis.

Principal component	Resistance rate (%)						Accumulative contribution rate (%)
	POD	APX	CAT	SOD	β-1,3-glucanase	MDA	
1	0.963	0.887	0.768	0.478	0.040	0.051	42.854
2	0.050	-0.349	0.456	0.126	0.952	-0.027	63.378
3	-0.065	-0.060	0.118	0.182	-0.035	0.981	80.177

*POD, peroxidase; APX, ascorbate peroxidase; CAT, catalase; SOD, superoxide dismutase; MDA, malonaldehyde.*

### Evaluation of Smut Resistance in Nine Sugarcane Genotypes by Subordinate Function Values

The comprehensive evaluation value (D) reflects cultivar resistance, and a high D value indicates a high level of resistance. The above-mentioned six physiological and biochemical indices were analyzed by subordinate function values and comprehensive evaluation values. **Table 5** shows that the levels of smut resistance in nine genotypes were in the following (high to low) order: YZ03-258 > FN40 > YZ01-1413 > GT02- 467 > ROC22 > YZ03-103 > YT96-86 > FN39 > LC05-136. The comprehensive evaluation values of YZ03-103 (0.24) and YT96-86 (0.23) were almost equal and these results were consistent with the ascending order of the copy numbers of smut pathogens at 7 days after inoculation (YZ03-258 < FN40 < YZ01-1413 < GT02-467 < ROC22 < YT96-86 < YZ03-103 < FN39 < LC05-136). Similarly, except for YT96-86, LC05-136, and FN40, the D value of the remaining sugarcane genotypes was consistent with the incidence of field smut disease (YZ03-258 < YZ01-1413/LC05-136 < YT96-86 < GT02-467 < ROC22 < FN39 < YZ03-103 < FN40).

**Table 5:** Subordinate function values of principal component and comprehensive evaluation of smut disease resistance in different sugarcane varieties under *Sporisorium scitamineum* stress.

Variety	Subordinate function values (*U*)			Integrated assessment value (*D*)	Order of smut disease resistance
	*U*(1)	*U*(2)	*U*(3)		
YZ03-258	1.00	0.85	0.32	0.82	1
YZ01-1413	0.56	0.48	1.00	0.63	3
YT96-86	0.27	0.00	0.43	0.23	7
LC05-136	0.00	0.32	0.38	0.16	9
GT02-467	0.84	0.01	0.03	0.46	4
ROC22	0.54	0.27	0.00	0.36	5
FN39	-0.12	1.00	0.09	0.21	8
YZ03-103	0.19	0.17	0.47	0.24	6
FN40	0.77	0.67	0.38	0.66	2

*U(1), *U*(2), and *U*(3) indicated the subordinate function value of principal components 1, 2, and 3, respectively.*

## Discussion

Sugarcane smut is a fungal disease that poses a serious threat to sugarcane production. Numerous studies have shown that use of resistant cultivars is the most cost-effective method for disease control (Xu and Chen, 2000; Scortecci et al., 2012; Sundar et al., 2012). Sugarcane smut invades tissues mainly by infecting the growing point of bud to produce a black whip-like structure with distinct morphological characteristics. Smut disease has a long latent infection period, and early smut pathogen infection in sugarcane seedlings and stems is not easily recognized based on morphological symptoms. Therefore, the establishment of a rapid detection system for the smut pathogen is important for the early diagnosis of this disease and breeding of resistant cultivars (Su et al., 2013a; Shen et al., 2016).

### TaqMan qRT-PCR Detection of Smut Pathogen Proliferation

As reported, the advent of microscopy (Nallathambi et al., 1998), conventional PCR amplification (Schenck, 1998), and enzyme-linked immunosorbent assay (ELISA; Naik et al., 2002) have aided development of detection techniques for sugarcane smut. With regard to PCR detection of sugarcane smut, Albert and Schenck (1996) designed a specific primer pair, bE4/bE8, for PCR detection of sugarcane smut pathogens. Singh et al. (2004) detected smut pathogens in sugarcane seedlings at 12 h after inoculation using the bE4/bE8 primer, whereas the phenotypic characteristics of smut in these seedlings were only expressed at 6 months after inoculation. However, the limitation of the conventional PCR assay is that it do not allow quantifying the amount of smut pathogen.

Quantitative diagnostics is important in study on the epidemiology of diseases, as they enable researchers to monitor fungal populations (Leisova et al., 2006). The qRT-PCR method with its high sensitivity, specificity, and precise quantification has been extensively applied to quantitative studies of gene expression, animal and plant pathogens (Jamnikar and Toplak, 2012; Li et al., 2012). The qRT-PCR technique has been successfully applied to the early detection of smut disease in *Tilletia controversa* (Nian et al., 2009), quantitative detection of soil *Rhizoctonia cerealis* (Sun et al., 2015), early detection of *Sclerotinia sclerotiorum* in rapeseed (Chen et al., 2011), early prediction of *S. sclerotiorum*, as well as rapid detection of *Phoma macdonaldii* in sunflower (Song et al., 2012). Previously, we have developed a TaqMan qRT-PCR detection system for smut pathogen (Su et al., 2013a). This system could detect the target pathogen in sugarcane samples at 12 h after inoculation with *S. scitamineum*. Significant differences in proliferation rates between two sugarcane genotypes were also observed. Whereas, a batch of sugarcane genotypes are required to validate this TaqMan assay for the evaluation of smut resistance.

In the present study, the TaqMan qRT-PCR technique was also used to determine the copy number of pathogens in nine sugarcane genotypes with different levels of smut resistance. The sugarcane cultivar specific differences in smut pathogen accumulation were found (**Figure 1**). At the early stage of smut inoculation (0–3 days), the proliferation rate of the smut pathogen was relatively low, and the proliferation rate peaked at 7 days post-inoculation. Except for YT96-86, LC05-136, and FN40, the copy number of smut pathogens in sugarcane genotypes was generally consistent with the field resistance levels. A higher level of field phenotypic resistance to smut resulted in a lower proliferation rate of this pathogenic fungi after smut inoculation. Similarly, Picó et al. (2003) described a relationship between the development of symptoms and the relative accumulation of cucumber vein yellowing virus (CVYV) in infected *Cucumis sativus* landraces. Moreover, Gil-Salas et al. (2009) have used nine commercial resistant and one non-resistant cucumber to compare the evolution of CVYV during spring and autumn seasons by TaqMan qRT-PCR assay and highlighted that this TaqMan method could be introduced into cucumber breeding programs as an improved resistance screening procedure. These findings were similar to those observed in our study that as an early detection technique for sugarcane smut disease, TaqMan qRT-PCR is highly feasible and has significant practical value.

### Physiological and Biochemical Indices Change Post Smut Pathogen Infection

Upon smut infection, the host plant undergoes a series of physiological and biochemical defense responses that are mainly achieved through the catalytic activity of defense enzymes (e.g., POD, SOD). The pathogen defense effectiveness depends on the speed and levels of these reactions at specific host sites (Blokhina et al., 2003). Due to ROS scavenging activity of defense enzymes, the intracellular ROS level is at equilibrium under normal circumstances. A rapid release of intracellular ROS can be triggered when the host is infected by a pathogen, thereby modifying the activity of the defense enzymes in each tissue. In addition, the defense mechanisms of the plant require the participation of many enzymes, thereby protecting the cells from further damage by the pathogen (Xu et al., 1994; Dat et al., 2000). In the present study, variations in the physiological and biochemical indices of nine genotypes in the presence of smut stress were observed (Supplementary Table S2), and the time points with the highest enzyme activity varied. This indicated that the sugarcane cultivars have different levels of potential smut resistance, which is consistent with the findings of Burner et al. (1993). The PCA method was used to convert the original six physiological and biochemical indices into three independent indicators (the first, the second, and the third principal components), with a cumulative contribution rate of 80.177% (**Table 4**). Therefore, it is possible to use these six physiological and biochemical indices for evaluation of sugarcane smut resistance. POD, APX, CAT, and SOD are important plant protective enzymes that remove H_2_O_2_, and can effectively prevent the accumulation of ROS (Xu et al., 1994; Sato et al., 2001; Xing, 2013; Su et al., 2014). As the first principal component, the contribution rate of these four enzymes was 42.856%. This primarily reflected the H_2_O_2_ scavenging capability in sugarcane under smut stress, and they were defined as removal factors of ROS. The second principal component was β-1,3-glucanase, which had a contribution ratio of 20.524%. β-1,3-glucanase catalyzes the hydrolysis of β-1,3-glucan and β-1,3-1,6-glucan, which are major components of fungal cell walls (Liu et al., 2010; Su et al., 2013b). Furthermore, the decomposition products can also induce the production of disease-related enzymes, promote the accumulation of disease-resistant material such as phytoalexin and lignin, and enhance the resistance of plants (Liu et al., 2010; Su et al., 2013b). Therefore, this principal component facilitates in the activation of the defense mechanism of sugarcane against the smut pathogen. The contribution rate of the third principal component, MDA, was 16.799%. MDA is a membrane lipid peroxidation end product and also an important indicator of the degree of the membrane system damage (Zhang B.Q. et al., 2015). It can be characterized as the degree of damage factor. Overall, the three comprehensive indices (POD, APX, CAT, and SOD), β-1,3-glucanase, and MDA, contained the majority of the information on the physiological and biochemical changes in the response to sugarcane to smut pathogen infection, and these can be used in the objective evaluation of resistance to smut disease at early cultivar selection stages.

Principal component analysis combined with subordinate function was used to evaluate the level of smut resistance of nine sugarcane genotypes, which were in the following (high to low) order: YZ03-258 > FN40 > YZ01-1413 > GT02-467 > ROC22 > YZ03-103 > YT96-86 > FN39 > LC05-136. These results are generally in agreement with the proliferation rates of smut pathogens for the nine genotypes, which were in the following (low to high) order: YZ03-258 < FN40 < YZ01-1413 < GT02-467 < ROC22 < YT96-86 < YZ03-103 < FN39 < LC05-136. Similarly, 310 somaclones from the sugarcane cultivar, CoC671, were dip-inoculated in a teliospore suspension, followed by a 2-years (plant and ratoon crops) identification of field resistance (Dalvi et al., 2012). Combined with conventional PCR, two sugarcane cultivars were screened. TC917 was identified as a smut-resistant cultivar and TC922 was a medium-smut-resistant cultivar. The yield of TC922 significantly greater than TC917 and its donor cultivar, CoC671. PCR analysis showed no proliferation of smut pathogen in the apical meristems of TC917 and TC922 during the growing season, from 2 months after inoculation to harvest time. However, the smut pathogen was detected during the 4th month in the susceptible cultivar, Co740. This result was consistent with the results of field resistance identification and indicated that PCR analysis could detect the proliferation of pathogens at an earlier growth stage. The measurement of resistance-related physiological and biochemical indices and the TaqMan qRT-PCR technique were used to evaluate sugarcane smut resistance based on physiological and biochemical changes and by detection of an increase in pathogen copy number. This approach helps elucidate the physiological and biochemical for smut resistance in sugarcane and also significantly shortens the time required for resistance identification.

Results on smut incidence in the field after early artificial dip-inoculation in the order (low to high) of YZ03-258 < YZ01-1413/LC05-136 < YT96-86 < GT02-467 < ROC22 < FN39 < YZ03-103 < FN40) and evaluation of the results of pathogen proliferation detection after indoor artificial puncture inoculation and physiological and biochemical indices were compared. LC05-136 and YT96-86 were medium susceptible and susceptible, respectively, although they were both scored as resistant types by field evaluation. Identification of disease resistance in plants can be performed by natural and artificial inoculation but the latter facilitates a better understanding of resistance under even high levels of pathogen stress. Byther and Steiner (1974) reviewed dip, puncture, germination, high-pressure spray, and other artificial inoculations. The dip method has been extensively applied in various countries for the evaluation of field resistance to sugarcane smut (Xu and Chen, 2000). Studies have shown that physiological, biochemical, and morphological changes contribute to the resistance of sugarcane to smut pathogen (Waller, 1970; Dean, 1982; Whittle and Walker, 1982; Xu and Chen, 2000). The bud scales protect the buds of some sugarcane cultivars against pathogen infection based on physical characteristics or biochemical characteristics. Under natural conditions, disease-resistant sugarcane cultivars with bud scale removal are more susceptible to smut after artificial inoculation (Waller, 1970; Benda and Koike, 1985; Padmanaban et al., 1988), indicating that smut resistance in sugarcane cultivars is relative. Pathogens introduced via puncture inoculation can cross the bud scale barrier and directly determine the physiological resistance of cultivars. Gillaspie et al. (1983) used the needle puncture inoculation technique to introduce smut pathogens into the bud body meristematic region of sugarcane cultivars and observed no onset of smut disease, indicating physiological resistance. We speculate that the test cultivars, YT96-86 and LC05-136, were morphologically resistant to smut, but lacked physiological resistance. This resulted in differences between the comprehensive evaluation result (medium susceptibility/susceptibility) in the indoor tests after needle puncture inoculation and the evaluation result (resistance) on the field after dip-inoculation. The relative nature of sugarcane smut resistance may also be influenced by regional differences. For example, a sugarcane cultivar with resistance to smut in one country or region may be susceptible to smut in another country or region because of genetic variations in the pathogen or differences in genotype-environment interactions (Singh et al., 2005). Burner et al. (1993) evaluated the potential resistance of 102 sugarcane cultivars using the methods of puncture inoculation and greenhouse cultivation. Different sugarcane cultivars exhibited a variety of physiological and biochemical resistances. The geographical environment had a relatively greater impact on the phenotypic resistance of sugarcane, and physiological and biochemical resistance remained intact compared to the morphological resistance observed during the breeding and screening processes (Burner et al., 1993). In this study, FN40, which was identified as smut susceptible in the field, was determined to be smut resistant indoors. This may be attributable to environmental differences.

## Conclusion

The TaqMan qRT-PCR assay and plant physiological and biochemical indices (POD, SOD, APX, CAT, MDA, and β-1,3-glucanase) were used to analyze the copy number of smut pathogens, as well as the physiological and biochemical changes in nine sugarcane genotypes with different levels of smut resistance after artificial inoculation. Except for LC05-136, YT96-86, and FN40, pathogen proliferation was in agreement with the results of physiological and biochemical indices, and these were generally in the same order as the incidences of field smut disease in the other six sugarcane genotypes (YZ03-258, YZ01-1413, GT02-467, ROC22, YZ03-103, and FN39). Cultivars with a high level of field phenotypic resistance to smut disease had relatively little pathogen proliferation after smut infection. These results illustrate an evaluation system for sugarcane smut that uses artificial puncture inoculation of sugarcane buds with smut pathogens, cultivation under indoor (or an incubator) controlled conditions, TaqMan qRT-PCR detection of pathogen proliferation and physiological and biological indices analysis at different time points after artificial inoculation. This method is rapid, accurate, and reliable, and is of significant potential value for the early identification of smut resistance in sugarcane.

## Author Contributions

LX conceived and designed the experiments. YS, ZW, QP, FL, and ZL performed the experiments. YS and ZW analyzed the data and wrote the paper. LX and YQ revised the paper. All authors read and approved the final version of the paper.

## Conflict of Interest Statement

The authors declare that the research was conducted in the absence of any commercial or financial relationships that could be construed as a potential conflict of interest.
