# Supplementary material for: Early Selection for Smut Resistance in Sugarcane Using Pathogen Proliferation and Changes in Physiological and Biochemical Indices
Source: Front Plant Sci. 2016 Jul 28;7:1133. doi: 10.3389/fpls.2016.01133 (PMC4963460; doi:10.3389/fpls.2016.01133)
Supplement: Supplementary file 2 [file Table_2.DOCX]

**Early selection for smut resistance in sugarcane using pathogen proliferation and changes in physiological and biochemical indices**

**Yachun Su^†^, Zhuqing Wang^†^, Liping Xu*, Qiong Peng, Feng Liu, Zhu Li, Youxiong Que***

Key Laboratory of Sugarcane Biology and Genetic Breeding, Ministry of Agriculture, Fujian Agriculture and Forestry University, Fuzhou, P. R. China

***Correspondence:** Liping Xu and Youxiong Que, Key Laboratory of Sugarcane Biology and Genetic Breeding, Fujian Agriculture and Forestry University, Ministry of Agriculture, No. 15 Shangxia Dian Road, Cangshan District, Fuzhou city, Fujian Province, 350000, P.R. China. E-mails: xlpmail@126.com, queyouxiong@126.com.

^†^These authors have contributed equally to this work.

**Supplementary Table S2 Physiological and biochemical indexes in 9 sugarcane varieties under *Sporisorium scitamineum* stress.**

| **Indexes** | **Inoculation times (d)** | **Variety** | | | | | | | | |
| --- | --- | --- | --- | --- | --- | --- | --- | --- | --- | --- |
|  |  | **YZ03-258** | **YZ01-1413** | **LC05-136** | **YT96-86** | **GT02-467** | **ROC22** | **FN39** | **YZ03-103** | **FN40** |
| POD  (U∙g^-1^∙FW∙min^-1^) | 0 | 3119.1±47.8 d | 4339.8±175.1 c | 2968.6±322.9 c | 3650.45±645.8 d | 5718.8±289.1 c | 5456.3±822.9 d | 4622.2±314.5 c | 5746.0±35.3 c | 4872.1±486.3 c |
|  | 1 | 7169.1±486.8 c | 5383.4±66.4 b | 2307.4±774.8 d | 8773.50±256.4 c | 7462.1±213.4 b | 9068.2±1065.4 b | 8502.3±169.0 a | 6842.5±171.5 b | 10840.7±90.8 b |
|  | 3 | 10894.0±256.0 a | 9215.9±293.8 a | 5155.1±99.6 a | 10768.33±569.4 b | 8766.0±1335.4 a | 6889.9±1089.5 c | 3911.0±362.5 c | 4269.6±151.2 d | 11933.5±216.2 ab |
|  | 7 | 8115.1±49.6 b | 9100.9±363.2 a | 4329.5±957.2 b | 14590.11±1881.4 a | 7452.9±59.9 b | 11163.2±786.4 a | 5693.2±118.8 b | 8857.5±252.0 a | 20476.2±82.4 a |
| APX  ( U∙g^-1^∙FW∙min^-1^) | 0 | 536.8±47.5 c | 775.7±77.6 c | 1497.3±54.7 a | 674.9±59.7 b | 572.4±151.8 d | 707.3±181.9 c | 2429.2±80.7 a | 1346.6±79.2 c | 818.3±30.5 c |
|  | 1 | 599.6±37.1 c | 812.5±102.6 c | 1150.9±49.5 c | 672.8±178.4 b | 856.8±57.5 c | 910.0±115.8 a | 2049.6±157.7 b | 1537.2±195.1 b | 1003.3±371.2 b |
|  | 3 | 724.9±83.2 b | 1223.2±81.2 a | 1287.7±53.6 b | 631.9±127.7 c | 1144.1±87.3 b | 839.4±54.0 b | 1318.4±26.6 d | 1130.1±113.2 d | 802.9±70.5 c |
|  | 7 | 1169.3±59.1 a | 1060.1±27.2 b | 1007.2±50.9 d | 1110.2±87.2 a | 1289.4±32.4 a | 914.2±55.4 a | 1431.2±103.3 c | 1916.7±54.2 a | 1304.0±152.8 a |
| CAT  ( U∙g^-1^∙FW∙min^-1^) | 0 | 27.2±0.9 d | 25.5±1.3 b | 33.0±2.5 c | 49.1±1.5 a | 30.9±1.7 c | 46.1±1.6 b | 19.4±2.4 c | 76.4±4.0 b | 23.1±1.7 d |
|  | 1 | 57.0±2.2 b | 28.2±2.2 b | 37.7±2.1 b | 38.7±1.2 b | 36.4±2.3 a | 26.6±3.1 d | 30.8±1.3 b | 103.6±3.3 a | 30.9±2.9 c |
|  | 3 | 75.7±1.7 a | 43.7±2.9 a | 31.1±2.0 c | 36.1±1.6 b | 31.7±3.2 c | 62.8±3.8 a | 53.9±2.8 a | 62.1±3.8 c | 41.2±2.3 a |
|  | 7 | 34.6±2.6 c | 41.3±2.2 a | 42.4±1.6 a | 28.5±2.1 c | 32.7±2.0 b | 32.3±2.7 c | 54.0±2.9 a | 52.1±3.0 d | 38.5±3.0 b |
| SOD  ( U∙g^-1^∙FW) | 0 | 94.9±0. 1 c | 115.3±0.2 d | 116.2±0.6 b | 148.6±0.3 a | 94.3±0.5 d | 121.3±0.5 c | 141.6±0.2 a | 130.4±0.2 a | 94.7±0.3 d |
|  | 1 | 141.0±0.2 b | 123.5±0.4 c | 126.0±0.5 a | 115.2±1.0 c | 141.7±0.4 b | 134.7±0.3 b | 85.8±0.3 c | 113.9±0.6 c | 104.4±0.3 c |
|  | 3 | 150.3±0.3 a | 140.6±0.3 a | 58.8±0.8 d | 108.5±1.3 d | 135.5±0.3 c | 142.7±0.5 a | 83.5±0.3 c | 122.6±0.4 b | 123.8±0.3 b |
|  | 7 | 135.9±0.3 ab | 135.5±0.1 b | 111.6±0.1 c | 138.1±0.2 b | 146.6±0.1 a | 117.9±0.3 d | 131.6±0.4 b | 105.5±0.2 d | 142.9±0.1 a |
| β-1,3- glucanase  ( U∙g^-1^∙FW∙min^-1^) | 0 | 15.4±0.7 b | 3.0±1.2 c | 38.3±2.4 b | 40.6±1.2 a | -23.8±2.4 d | 3.6±1.2 ab | -23.3±1.0 d | -24.1±1.0 a | -101.8±2.9 c |
|  | 1 | -1.9±1.6 c | 49.7±5.0 a | 51.7±0.1 a | 8.9±1.1c | -15.5±2.3 c | 1.9±0.9 b | -15.0±3.1 c | -28.9±4.7 b | -84.2±1.5 b |
|  | 3 | -10.9±3.2 d | 13.7±3.5 b | 30.6±1.1 c | 40.5±0.4 a | 27.4±2.2 a | -23.5±1.8 c | 10.1±1.8 b | -21.1±1.2 a | -46.8±1.1 a |
|  | 7 | 38.9±1.1 a | -23.0±1.8 d | 15.8±0.3 d | 26.6±1.4 b | 14.5±1.5 b | 6.8±2.9 a | 54.6±0.5 a | -56.3±0.7 c | -48.8±2.0 a |
| MDA  (mol∙g^-1^∙FW) | 0 | 2.3±0.3 d | 2.0±0.1 c | 6.0±0.3 a | 4.8±0.5 a | 4.0±0.5 b | 2.5±0.1 c | 5.4±0.9 a | 2.3±0.3 a | 1.8±0.1 d |
|  | 1 | 4.3±0.2 b | 1.5±0.7 d | 5.7±0.3 b | 2.1±0.4 c | 4.5±0.2 a | 4.3±0.1 a | 3.4±0.6 c | 2.3±0.7 a | 2.7±0.1 b |
|  | 3 | 4.7±0.9 a | 3.7±0.4 a | 2.1±0.6 d | 2.7±0.4 b | 3.8±0.9 c | 4.2±0.4 b | 3.1±0.8 d | 2.3±0.4 a | 2.0±0.5 c |
|  | 7 | 2.7±0.1 c | 2.5±0.4 b | 3.8±0.1 c | 2.0±0.8 d | 2.2±0.1 d | 1.7±0.9 d | 5.0±0.2 b | 1.3±0.4 b | 3.3±0.3 a |

Data followed different small letters in the same variety indicate significant difference at 0.05 level.
