# Supplementary material for: Early Selection for Smut Resistance in Sugarcane Using Pathogen Proliferation and Changes in Physiological and Biochemical Indices
Source: Front Plant Sci. 2016 Jul 28;7:1133. doi: 10.3389/fpls.2016.01133 (PMC4963460; doi:10.3389/fpls.2016.01133)
Supplement: Supplementary file 3 [file Data_Sheet_1.DOCX]

**Early selection for smut resistance in sugarcane using pathogen proliferation and changes in physiological and biochemical indices**

**Yachun Su^†^, Zhuqing Wang^†^, Liping Xu*, Qiong Peng, Feng Liu, Zhu Li, Youxiong Que***

Key Laboratory of Sugarcane Biology and Genetic Breeding, Ministry of Agriculture, Fujian Agriculture and Forestry University, Fuzhou, P. R. China

***Correspondence:** Liping Xu and Youxiong Que, Key Laboratory of Sugarcane Biology and Genetic Breeding, Fujian Agriculture and Forestry University, Ministry of Agriculture, No. 15 Shangxia Dian Road, Cangshan District, Fuzhou city, Fujian Province, 350000, P.R. China. E-mails: xlpmail@126.com, queyouxiong@126.com.

^†^These authors have contributed equally to this work.

**POD Enzyme Activity Measurement**

POD enzyme activity analysis was conducted based on the method described by Xing (2013) with minor modifications (**Supplementary File 1**). Five quartz cuvettes with a light path of 1 cm were used. Approximately 3 mL of the reaction solution was added to one cuvette as zero control; 3 mL of the reaction solution and 50 μL of the enzyme solution that was inactivated in boiling water bath for 15 min were added into one cuvette as blank control; 3 mL of the reaction solution and 50 μL enzyme solution were respectively added into three cuvettes as replicates. Measurement was continuously performed for 3 min at a wavelength of 470 nm after a 1 min reaction at room temperature, optical density (OD) values were recorded, and one enzyme activity unit was expressed as a 0.1 unit change in absorbance per minute.

**SOD Enzyme Activity Measurement**

SOD enzyme activity analysis was conducted based on the method of Mo (2012) with minor modifications. The reaction solution (2.3 mL) contained 0.3 mL of 0.05 mol/L phosphate buffer (pH 7.8), 0.3 mL of 0.13 mol/L methionine solution, 0.3 mL of 0.75 × 10^−3^ mol/L nitroblue tetrazolium (NBT) solution, 0.3 mL of 0.1 × 10^−3^ mol/L ethylenediamine tetraacetic acid disodium salt (EDTA-Na_2_), and 0.2 mL of purified water. Five quartz cuvettes with a light path of 1 cm were used, of which two served as control tubes, whereas the other three contained 2.3 mL of the reaction solution, 0.3 mL of 0.02 × 10^−3^ mol/L riboflavin, and 0.1 mL of enzyme solution (a phosphate buffer with a pH of 7.8 used for control tube), which were successively added. After the samples were thoroughly mixed, one control tube was placed in the dark, and the rest tubes were placed in an incubator with a light intensity of 4,000 lx for 20 min at a constant temperature. After incubation, the control that was placed in the dark was used as blank, and the absorbance of all other tubes were measured at a wavelength of 560 nm. One enzyme activity unit was expressed as 50% inhibition of NBT photoreduction.
